# Supplementary material for: Overproduction of Human Zip (SLC39) Zinc Transporters in Saccharomyces cerevisiae for Biophysical Characterization
Source: Cells. 2021 Jan 21;10(2):213. doi: 10.3390/cells10020213 (PMC7911073; doi:10.3390/cells10020213)
Supplement: Supplementary file 1 [file cells-10-00213-s001.pdf]

Overproduction of human ZIP (SLC39) zinc transporters in *Saccharomyces cerevisiae* for biophysical characterization

Supplementary Material

Table S1

Table S1. Sequences of cloning primers used to engineer expression constructs of human Zrt-, Irt-like proteins (hZIPs) described in the study. Targets are shown in the colors corresponding to the main text.

Construct	Sequence (5'–3')
**TEV-GFP_FW**	AAAATTTGTATTTTCAAAGTCAATTTTCTAAAGGTGAAGAATTATTCACT
**GFP-His_RV**	CTTCAATGCTATCATTTCCTTTGATATTGGATCATCTAATGGTGATG GTGATGGTGATGGTGTTTGTACAATTCA
**hZIP1_FW**	ACACAAATACACACACTAAATTACCGGATCAATTCTAAGATAATTATGGGTC CATGGGGTGAAC
**hZIP1-TEV-GFP-His_RV**	AAATTGACTTTGAAAATACAAATTTTCTATTTGAATGAACAACAAACC
**His-TEV-hZIP1_FW**	ACACAAATACACACACTAAATTACCGGATCAATTCTAAGATAATTATGCACC ATCACCATCACCATCACCATGAAAATTTGTATTTTCAAAGTATGGGTCCATGG GGTGAACC
**StrepII-TEV-hZIP1_FW**	ACACAAATACACACACTAAATTACCGGATCAATTCTAAGATAATTATGGCAA GCTGGAGCCACCCGCAGTTCGAAAAGGGTGCAGAAAATTTGTATTTTCAAAG TATGGGTCCATGGGGTGAACC
**hZIP1_RV**	CTTCAATGCTATCATTTCCTTTGATATTGGATCATTTATATTTGAATGAACAA CAAACCTG
**hZIP2_FW**	ACACAAATACACACACTAAATTACCGGATCAATTCTAAGATAATT ATGGGTCCATGGGGTGAAC
**hZIP2-TEV-GFP-His_RV**	AAATTGACTTTGAAAATACAAATTTTCTGCCCATAAAGCTATAAAGGC
**hZIP11_FW**	ACACAAATACACACACTAAATTACCGGATCAATTCTAAGATAATTATGTTGC AAGGTCATTCTTCAG
**hZIP11-TEV-GFP-His_RV**	AAATTGACTTTGAAAATACAAATTTTCACCTAAACCGACATCCAATGACAT
**hZIP13_FW**	ACACAAATACACACACTAAATTACCGGATCAATTCTAAGATAATTATGCCAG GTTGTCCATGCC
**hZIP13-TEV-GFP-His_RV**	AAATTGACTTTGAAAATACAAATTTTCATCTACGAACAATGAAAACAA

TEV: cleavage site for tobacco etch virus protease
GFP: green fluorescent protein
His: octa-histidine-tag
StrepII: StrepII-tag

Table S2

Table S2. Solubilization conditions and buffers used during affinity purification and size-exclusion chromatography of the respective hZIP1 fusions.

Construct	Solubilization buffer	Solubilization detergent and duration (at 4 °C)	Affinity chromatography elution buffer	Size-exclusion chromatography buffer
**hZIP1-TEV-GFP-His**	50 mM Tris-HCl pH 8.0<br>200 mM NaCl<br>30 % glycerol<br>5 mM BME<br>1 mM PMSF<br>SigmaFAST™ protease inhibitor cocktail	2 % DDM + 0.68 % CHS<br>2 h	20 mM Tris-HCl pH 8.0<br>200 mM NaCl<br>20 % glycerol<br>5 mM BME<br>0.05 % DDM + 0.017 % CHS<br>imidazole (linear gradient 25-500 mM)	not applicable
**His-TEV-hZIP1**	20 mM Tris-HCl pH 7.0<br>200 mM NaCl<br>20 % glycerol<br>0.5 mM EDTA<br>0.5 mM EGTA<br>5 mM BME<br>1 mM PMSF<br>1 μg L^{-1} chymostatin<br>1 μg L^{-1} leupeptin<br>1 μg L^{-1} pepstatin	2 % DDM + 0.2 % CHS<br>2 h	20 mM Tris-HCl pH 7.0<br>200 mM NaCl<br>20 % glycerol<br>5 mM BME<br>0.05 % DDM + 0.005 % CHS<br>imidazole (linear gradient 50-500 mM)	20 mM MES-NaOH pH 6.0<br>100 mM NaCl<br>10 % glycerol<br>2 mM BME<br>0.03 % DDM + 0.003% CHS
**StrepII-TEV-hZIP1**	20 mM Tris-HCl pH 8.0<br>200 mM NaCl<br>20 % glycerol<br>0.5 mM EDTA<br>0.5 mM EGTA<br>5 mM BME<br>1 mM PMSF<br>1 μg L^{-1} chymostatin<br>1 μg L^{-1} leupeptin<br>1 μg L^{-1} pepstatin	2 % DDM + 0.2% CHS<br>2 h	20 mM Tris-HCl pH 8.0<br>200 mM NaCl<br>20 % glycerol<br>5 mM BME<br>0.05 % DDM + 0.005 % CHS<br>biotin (pulse elution with 50 mM)	20 mM MES-NaOH pH 5.0<br>100 mM NaCl<br>10 % glycerol<br>2 mM BME<br>0.03 % DDM + 0.003% CHS

TEV: cleavage site for tobacco etch virus protease
GFP: green fluorescent protein
His: octa-histidine-tag
BME: β-mercaptoethanol
CHS: cholesteryl hemisuccinate Tris salt
StrepII: StrepII-tag

Figure S1

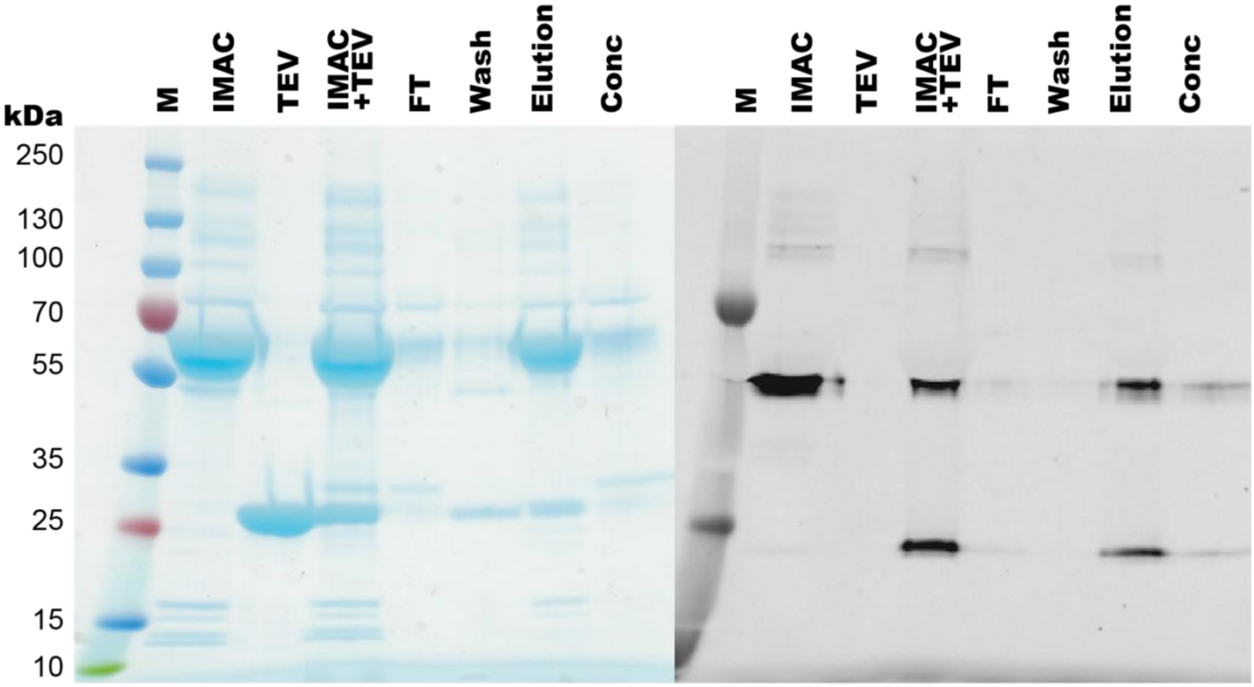

Figure S1. Cleavage of hZIP1-TEV-GFP-His fusion with tobacco etch virus (TEV) protease. Coomassie staining (left) and in-gel GFP fluorescence (right) of the corresponding SDS-PAGE-separated samples collected during TEV protease digestion of immobilized metal affinity chromatography (IMAC)-pure hZIP1-TEV-GFP-His fusion (IMAC purification shown in Fig. 4). TEV protease treatment was performed for 16 h at 4 °C, followed by reverse (R)-IMAC to rebind released TEV-GFP-His tag together with His-tagged TEV protease. M: marker; IMAC: IMAC-pure hZIP1-TEV-GFP-His; TEV: TEV protease alone; IMAC+TEV: IMAC-pure hZIP1-TEV-GFP-His cleaved with TEV protease; FT: R-IMAC flow-through; Wash: R-IMAC wash with 100 mM imidazole, Elution: R-IMAC elution with 300 mM imidazole; Conc: concentrated R-IMAC FT. The predicted MWs of the respective forms are: 63.3 kDa (hZIP1-TEV-GFP-His), 32.5 kDa (hZIP1), 28.0 kDa (TEV-GFP-His) and 29.5 kDa (TEV-His). Only a minor fraction of the protein sample elutes in the R-IMAC FT, indicating very low TEV protease cleavage efficiency of hZIP1-TEV-GFP-His fusion.

Figure S2

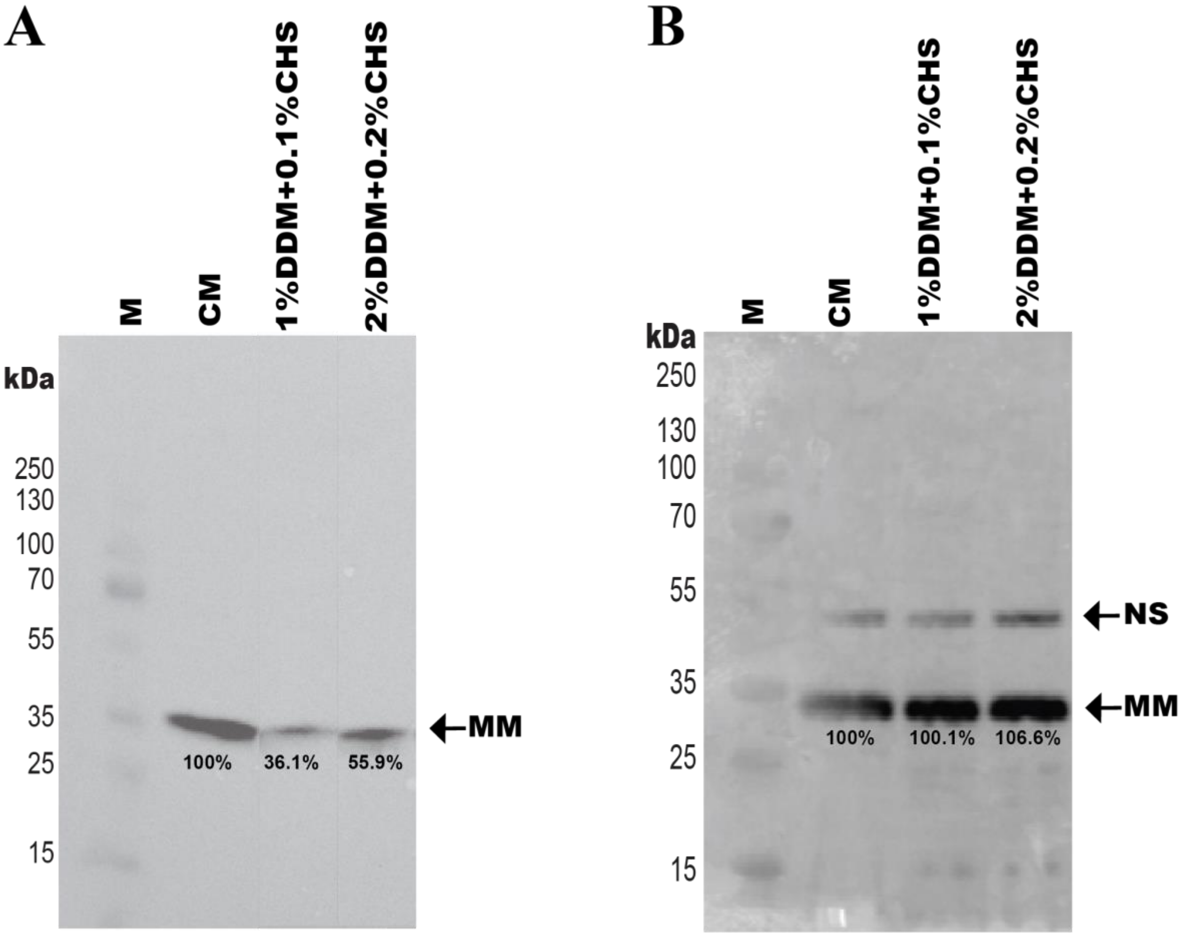

Figure S2. Detergent screening of N-terminal hZIP1 fusions. Crude membranes isolated from *Saccharomyces cerevisiae* cells (derived from 12-L cultures) expressing **(A)** His-TEV-hZIP1 and **(B)** StrepII-TEV-hZIP1 (68-h induction at 15 °C) were solubilized for 120 min at 4 °C with increasing final concentrations of DDM supplemented with CHS (1 % + 0.1 %, and 2 % + 0.2 %, respectively). Following solubilization, the supernatant after ultracentrifugation was analyzed. CHS: cholesteryl hemisuccinate Tris salt. **(A)** Immunoblot of SDS-PAGE-separated detergent-solubilized crude *S. cerevisiae* membranes overexpressing His-TEV-hZIP1 probed with 6×His mAb-HRP conjugate. M: marker; CM: crude membranes. Arrow indicates the monomeric (MM) form of the construct, with the predicted MW of 36.3 kDa. **(B)** Immunoblot of SDS-PAGE-separated detergent-solubilized crude *S. cerevisiae* membranes overexpressing StrepII-TEV-hZIP1 probed with Strep-Tactin® HRP conjugate. M: marker; CM: crude membranes. Arrows indicate the monomeric (MM) form of the construct, with the predicted MWs of 36.5 kDa and the non-specific band (NS) with higher electrophoretic mobility (~50 kDa). Numbers below each lane represent normalized signal intensities (%) relative to the signal from CM, as quantified using ImageJ software (https://imagej.nih.gov/ij/).

Figure S3

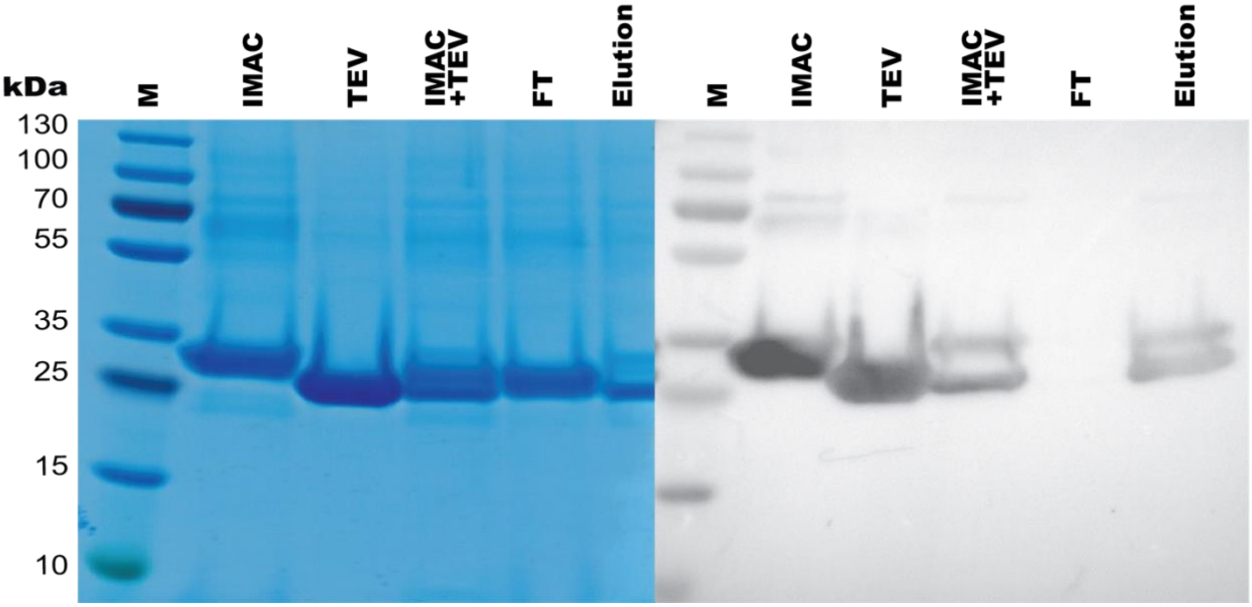

Figure S3. Cleavage of His-TEV-hZIP1 fusion with tobacco etch virus (TEV) protease. Coomassie staining (left) and immunoblot probed with 6×His mAb-HRP conjugate (right) of the corresponding SDS-PAGE-separated samples collected during TEV protease digestion of immobilized metal affinity chromatography (IMAC)-pure His-TEV-hZIP1 fusion (IMAC purification shown in Figs. 5A and B). TEV protease treatment was performed for 16 h at 4 °C, followed by reverse (R)-IMAC to rebind released His-TEV tag together with His-tagged TEV protease. M: marker; IMAC: IMAC-pure His-TEV-hZIP1; TEV: TEV protease alone; IMAC+TEV: IMAC-pure His-TEV-hZIP1 cleaved with TEV protease; FT: R-IMAC flow-through; Elution: R-IMAC elution with 300 mM imidazole. The predicted MWs of the respective forms are: 34.3 kDa (His-TEV-hZIP1), 32.5 kDa (hZIP1), 2.0 kDa (His-TEV) and 28.0 kDa (TEV-His). A major fraction of the protein sample elutes in the R-IMAC FT, indicating very high TEV protease cleavage efficiency of His-TEV-hZIP1 fusion.
